# Effects of Temperature on Lifespan of *Drosophila melanogaster* from Different Genetic Backgrounds: Links between Metabolic Rate and Longevity

**DOI:** 10.3390/insects11080470

**Published:** 2020-07-25

**Authors:** Mateusz Mołoń, Jan Dampc, Monika Kula-Maximenko, Jacek Zebrowski, Agnieszka Mołoń, Ralph Dobler, Roma Durak, Andrzej Skoczowski

**Affiliations:** 1 Department of Biochemistry and Cell Biology, University of Rzeszow, 35-601 Rzeszów, Poland; 2 Department of Experimental Biology and Chemistry, University of Rzeszów, 35-310 Rzeszów, Poland; dampcjan@gmail.com (J.D.); agnieszkamaslowska@o2.pl (A.M.); rdurak@univ.rzeszow.pl (R.D.); 3 The Franciszek Górski Institute of Plant Physiology, Polish Academy of Sciences, 30-239 Krakow, Poland; kula.monika@gmail.com; 4 Department of Plant Physiology and Ecology, University of Rzeszow, 35-310 Rzeszów, Poland; jaze28@interia.pl; 5 Applied Zoology, Faculty of Biology, TU Dresden, 01062 Dresden, Germany; ralph.dobler@tu-dresden.de; 6 Institute of Biology, Pedagogical University of Krakow, 30-084 Krakow, Poland; andrzej.skoczowski@up.krakow.pl

**Keywords:** aging, fruit fly, lifespan, microcalorimetry

## Abstract

Despite many studies of the aging process, questions about key factors ensuring longevity have not yet found clear answers. Temperature seems to be one of the most important factors regulating lifespan. However, the genetic background may also play a key role in determining longevity. The aim of this study was to investigate the relationship between the temperature, genetic background (fruit fly origin), and metabolic rate on lifespan. Experiments were performed with the use of the wild type *Drosophila melanogaster* fruit flies originating from Australia, Canada, and Benin and the reference OregonR strain. The metabolic rate of *D. melanogaster* was measured at 20 °C, 25 °C, and 28 °C in an isothermal calorimeter. We found a strong negative relationship between the total heat flow and longevity. A high metabolic rate leads to increased aging in males and females in all strains. Furthermore, our results showed that temperature has a significant effect on fecundity and body weight. We also showed the usefulness of the isothermal calorimetry method to study the effect of environmental stress conditions on the metabolic activity of insects. This may be particularly important for the forecasting of impact of global warming on metabolic activity and lifespan of various insects.

## 1. Introduction

Aging is defined as any age-specific decline in traits associated with individual fitness; specifically, an increased mortality and lower reproduction and physiological performance [1] has been viewed as a natural process [2,3]. Aging is also a universal process that has been identified in organisms ranging from *Prokaryotes* to *Eukaryotes* [4]. To gain knowledge about basic biological phenomena, such as senescence, studies on model organisms are used, ranging from single-cell yeast, through the nematode and fruit fly, to the rat and mouse. In this study, we focused on the fruit fly *Drosophila melanogaster* to investigate aging.

The fruit fly is one of the most commonly used model organisms to study aging, as it has many benefits (e.g., short lifespan, completely sequenced genome, simple strain maintenance, ease of environmental and genetic manipulations to affect its lifespan, and powerful molecular techniques) [4]. It has, therefore, a long tradition in aging research, initiated by Loeb and Northrop [5] in 1916. As with other model organisms, many genetic and non-genetic factors may affect the lifespan of *D. melanogaster*. Among genetic manipulations are many pathways associated with oxidative stress defense [6,7], DNA repair [8], single gene expression [9], and Insulin/IGF-like signaling [10]. On the other hand, ambient temperature, mild stress, and diet are often discussed as non-genetic factors determining aging [11,12]. The reproductive status may also affect lifespan. Virgin *Drosophila* females have their lifespans extended twice in comparison to mated females [13]. In turn, Partridge and Farquhar [14] showed that male virgins also live longer which led to the conclusion that sexual activity reduces the lifespan of male fruit flies. Dietary status may determine lifespan in two ways. On the one hand, the length of life may be extended by dietary or caloric restriction; on the other hand, it may be reduced by a fat-rich diet [14,15]. Also, many biologically active compounds (e.g., naringenin or curcumin) determine lifespan [15,16]. Temperature may also affect lifespan, presumably by changing energy status [17]. It has been long speculated that it may influence aging due to the more molecular damage being generated [18].

The longevity of animals depends on the metabolic rate according to what is known as the rate of metabolism theory [19]. In this context, longevity is inversely proportional to the level of the basal metabolic rate measured as oxygen consumption. This theory is connected with mammals [20] but is also supported by studies on *D. melanogaster* [17] and yeast [21]. Fruit flies as poikilothermic organisms live shorter lifespans at higher temperatures because their metabolic rate is higher [17]. Similarly, aphids have shorter lifespans at higher temperatures [22,23]. More recent data showed that an increase in temperature to 28 °C increased metabolism in *Aphis pomi* five-fold, measured as heat energy flow, which caused shortened aphid longevity [24]. The effect of temperature on insect metabolism has been investigated in many species. Correlation between temperature and metabolic rate was shown previously in studies on pupae of *Platynota stultana* [25,26], *Musca domestica* [27], and *Cydia pomonella* [28]. Interestingly, the metabolic rate resulting from temperature changes may depend on species or geographical coverage. A good example is the comparison of metabolic activity of the Egyptian bee *Apis mellifera lamarckii* and the European *Apis mellifera carnica*. Both of these populations show a different way of reacting to increases in the temperature. The European population at high temperatures show slight changes in calorimetric measurement, while the African population shows a reduction in the heat flow [29]. Similarly, in unicellular organisms more intense biosynthesis and reproduction processes generate a higher metabolic rate, resulting in a reduction of lifespan [21]. As shown by the previous data, temperature can modulate lifespan in unicellular organisms without affecting their fertility [30]. Additionally, genetic background may play a key role in determining lifespan [31,32]. It is yet unknown which of these two factors is more important in determining longevity: that is environmental conditions, such as temperature, or genetic background. Therefore, the aim of this study was to investigate the relationship between temperature (fruit fly origin) and metabolic activity of the fruit fly measured as a quantity of the produced heat. We measured the amount of the heat produced by flies using a new generation isothermal calorimeter which allowed for performing measurement in a non-invasive manner. Here, we also asked whether the fruit fly’s origins have an impact on aging.

## 2. Materials and Methods

### 2.1. Fly Stock and Rearing

Experiments were performed on the wild-type *D. melanogaster* fruit flies originating from Australia, Canada, and Benin, and the OregonR strain (Bloomington Drosophila Stock Center). 

The population from Australia had originally been sourced from Coffs Harbour and was collected in 2010 [33]. The population from Benin, Africa, was originally collected in 1970, is routinely used for *Drosophila* genetics research, and is called the Dahomey population [34,35]. The population from Dundas, Canada, was originally collected in 2005 [36].

Flies were kept in the laboratory in 100 mL bottles at 20 °C, 25 °C, and 28 °C; the photoperiod was 12/12 h (day/night, respectively) with 60% relative humidity on standard fly medium consisting of brewer’s yeast (80 g/L), sucrose (50 g/L), agar (15 g/L), and Nipagin (Sigma–Aldrich) (8 mL/L). To generate the flies for the experiments, groups of 10 mated females were allowed to lay eggs in 100 mL rearing bottles during a restricted period of 6 h maximum under their common laboratory conditions. No CO_2_ anesthesia was used, as it affects metabolic traits [37].

### 2.2. Measurement of Lifespan in Drosophila melanogaster

To measure the lifespan of the flies, virgin flies separated by sex were transferred into vials containing a standard diet as described elsewhere [38]. Flies lived under standard conditions (humidity and light/dark rhythm) at three different temperatures: 20 °C, 25 °C, and 28 °C. For each sex and line, 10 replicate vials were set-up with 30 flies in each vial. Flies were transferred with CO_2_ anesthesia to vials with fresh food every two days, during which the number of dead flies was recorded. Flies continued to be transferred until all flies were dead. 

### 2.3. Measurement of Fly Fecundity

Virgin flies were kept at two males and two virgin females on a standard diet as described elsewhere [11]. Flies were transferred to fresh medium every day, and the number of laid eggs was counted every day for 15 days. For each temperature, 10 replicates were set-up for each fly strain.

### 2.4. Body Weight

Newly emerged flies were separated by sex and allowed to feed on standard medium for 15 days at each temperature. Flies were then subjected to mild etherisation followed by immediate measurement of body weight in batches of 10 flies. For each temperature, 100 flies were measured for each fly strain and sex.

### 2.5. Determination of the Heat Flow Using Isothermal Calorimetry

Metabolic activity of *D. melanogaster* was measured at 20 °C, 25 °C, and 28 °C in a TAM III isothermal calorimeter equipped with TAM Assistant Software (TA Instruments, Lindon, UT). Five newly emerged flies of each strain, male and female separately, were placed in 4.0 mL calorimetric ampoules with 0.5 mL of agar with apple juice. After the 30 min needed for balancing of temperature, the the heat flow (ϕ curves) was recorded (in µW) over 24 h. The specific heat flow–time plots (ϕ–t) were obtained by normalization of the particular curves to the mass of the flies (µW mg^−1^). The metabolic activity of *D. melanogaster* was determined by integrating the heat flow curves over a time interval of 24 h to give the total heat flow (TQ-metabolic activity) in joules per gram of body weight of flies and per hour (J g^−1^ h^−1^). The data presented are the average values of 9 independent repetitions for each object. The degree of impact of the origin of flies on thermal energy production in individual common rearing temperatures was determined (separately for males and females) using the two-way ANOVA (*p* ≤ 0.05). 

### 2.6. Statistical Analysis

The survival analysis [39,40] was used to analyze longevity data. Kaplan–Meier curves with 95% confidence intervals were calculated and plotted for non-censored data using the package *survival* in R. The log-rank test (chi-square statistic) [41] was applied with the same weight to all events to assess whether overall differences existed among the survival curves. The effects of temperature and the place of origin were analyzed as described below. Because of violation of the normality and homoscedasticity assumptions (Shapiro–Wilk’s and Bartlett tests, respectively) as well as due to the presence of outliers, the statistical tests were performed with robust two-way ANOVA methods for medians.

The pbad2way (2000 bootstrap samples) and the med2way functions (R, package *WRS2*) [42] were used to test the equality of medians across various groups (origin, temperature) as well as to estimate the interactions between both factors. In the case of rejection of the null hypothesis of equal medians, a robust post-hoc test (pairwiseRobustMatrix function, *WRS2* package in R) was employed for pairwise comparison of the median of the groups. Linear regression analysis (heat energy versus lifespan) was accomplished with the panel.quantile function (latticeExtra in R). The data are presented in figures mostly as box plots [43] with median value (horizontal line), 95% confidence interval of the median (the notch), and the interquartile range containing 50% of data (colored areas). All figures present raw data. The outliers are marked with different colors. A significance level of 0.05 was assumed for the statistical hypothesis testing and the pairwise group comparison. The statistical computing and most of data presentation were performed using R programming.

## 3. Results

### 3.1. Temperature Effects on Longevity

To examine the impact of temperature on lifespan in different genetic backgrounds, we used fly populations originated from Australia, Benin, and Canada and the widely used laboratory OregonR strain. To investigate potential dependencies between the genetic background and temperature, we used three different temperature conditions. We used the temperature of 25 °C as the laboratory optimum for fruit fly life, 20 °C as a low temperature, and 28 °C as a heat stress. First, we analyzed lifespan in different temperatures according to the standard procedure. We found a significant effect of temperature on the maximum lifespan and mean lifespan (*p* < 0.05). In general, the high temperature (28 °C) caused a decrease in lifespan for all strains and both sexes (Table S1). 

**Figure 1:**
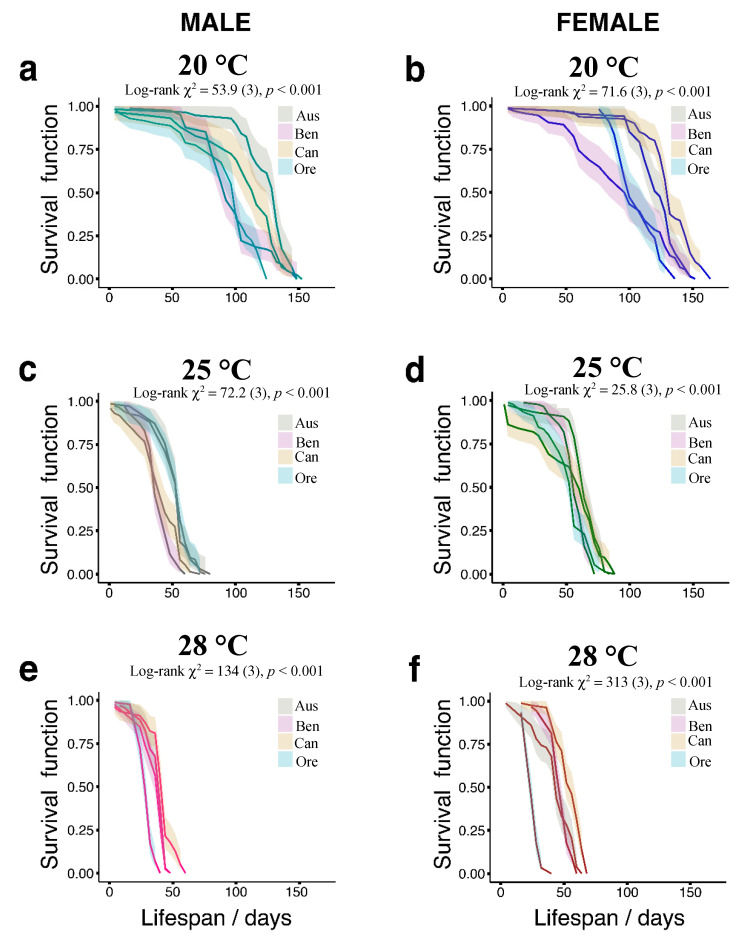
Survival curves showing the origin and temperature modulation of longevity in *Drosophila melanogaster*. Lifespans of adult male and female fruit flies fed a standard diet and grown at 20 °C (**a**,**b**), 25 °C (**c**,**d**), and 28 °C (**e**,**f**). Filled colored ribbons show 95% confidence intervals. The log-rank test (chi-squared statistics) was used to evaluate whether differences existed among overall curves and to provide *p*-values. For each sex, 10 replicate vials were set up with 30 flies in each vial. Aus—Australian population; Ben—Benin population; Can—Canadian population; Ore—OregonR laboratory strain.

**Figure 2:**
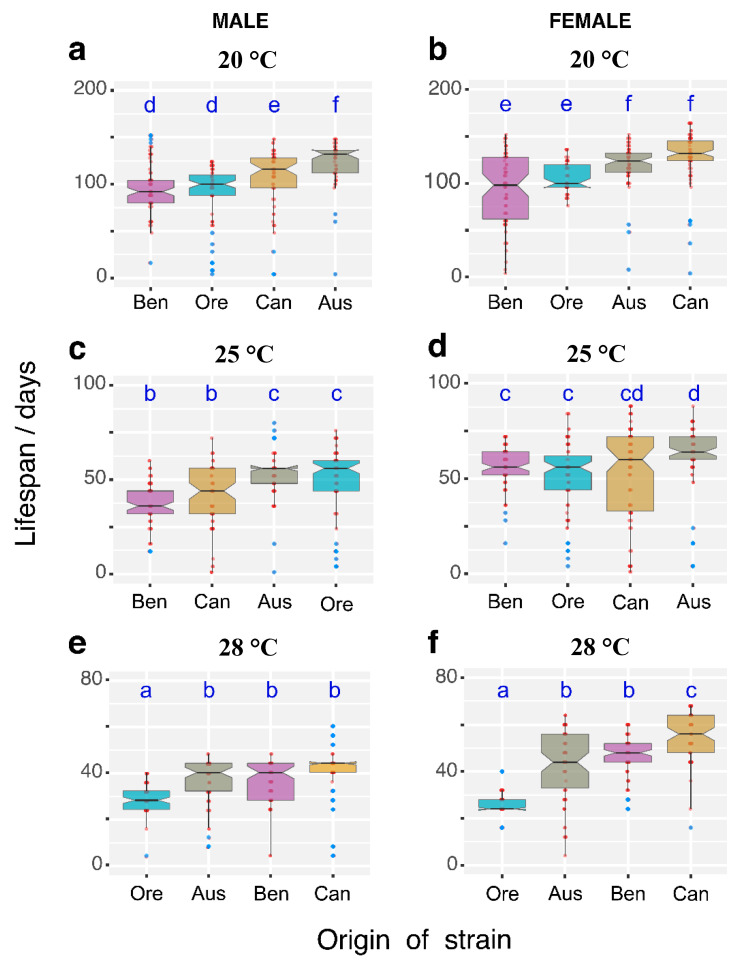
Lifespans of adult male and female flies fed a standard diet and grown at 20 °C (**a**,**b**), 25 °C (**c**,**d**), and 28 °C (**e**,**f**). The box plots (median, interquartile range, and 95% confidence interval) describe variability of the strains in the order of increasing median values. Robust two-way ANOVA (pbad2way function, R package *WRS2*) was followed by a robust post-hoc test (pairwise Robust Matrix function, *WRS2* package in R) for pairwise comparison of the median of groups. Statistical (*p* < 0.05) differences among the groups of various origin (across all the temperature levels) are indicated by different letters. The letters are alphabetically ordered according to the ascending median values. The outliers in the plot are marked by the shaded squares. The measurements (10 replicate vials with 30 flies in each vial) and statistical analysis were performed separately for each sex. Aus—Australian population; Ben—Benin population; Can—Canadian population; Ore—OregonR laboratory strain.

Low temperature conditions generally caused an increase in longevity. Differences among strains with different origins were most pronounced at 20 °C for both sexes (Figure 1a,b). At 20 °C, the mean lifespan of Australian males was significantly higher compared to other male genotypes (*p* < 0.05). The Canadian flies showed a slightly shorter mean lifespan, while the values for the Benin and OregonR strains were comparatively low (Figure 2a) (*p* < 0.05). Among the female genotypes, the Canadian and Australian flies had the longest lifespans, while the Benin and OregonR flies had the shortest lifespans (*p* < 0.05). For males and females, the Australian and the Canadian flies had the longest lifespans, while flies from Benin and OregonR had the shortest lifespans.

At the optimum rearing temperature of 25 °C, the shape of the survival curves among the fly populations were more consistent than at 20 °C. It is likely that the temperature effect begins to be more pronounced, while the effect of the genotype is less evident. At 25 °C, males from Australia and Oregon had the longest lifespan, while we observed the shortest lifespan for the Benin and Canada males (Figure 1c and Figure 2c) (*p* < 0.05). We observed the same pattern for females with the longest lifespan for the Australian flies and the shortest lifespan for the Benin and OregonR flies (Figure 1d and Figure 2d) (*p* < 0.05). At the highest temperature of 28 °C, in the male genotype, the shortest lifespan was recorded for the OregonR strains, while the longest were for the strains originating from Canada, Benin, and Australia (Figure 1e and Figure 2e) (*p* < 0.05). As for the males, we found that the OregonR females had the shortest lifespan, and the Canadian females had the longest lifespan (*p* < 0.05). The Australian and Benin females lived longer than the OregonR females but shorter than the Canadian females (Figure 1f and Figure 2f).

### 3.2. Temperature Effect on Fertility

**Figure 3:**
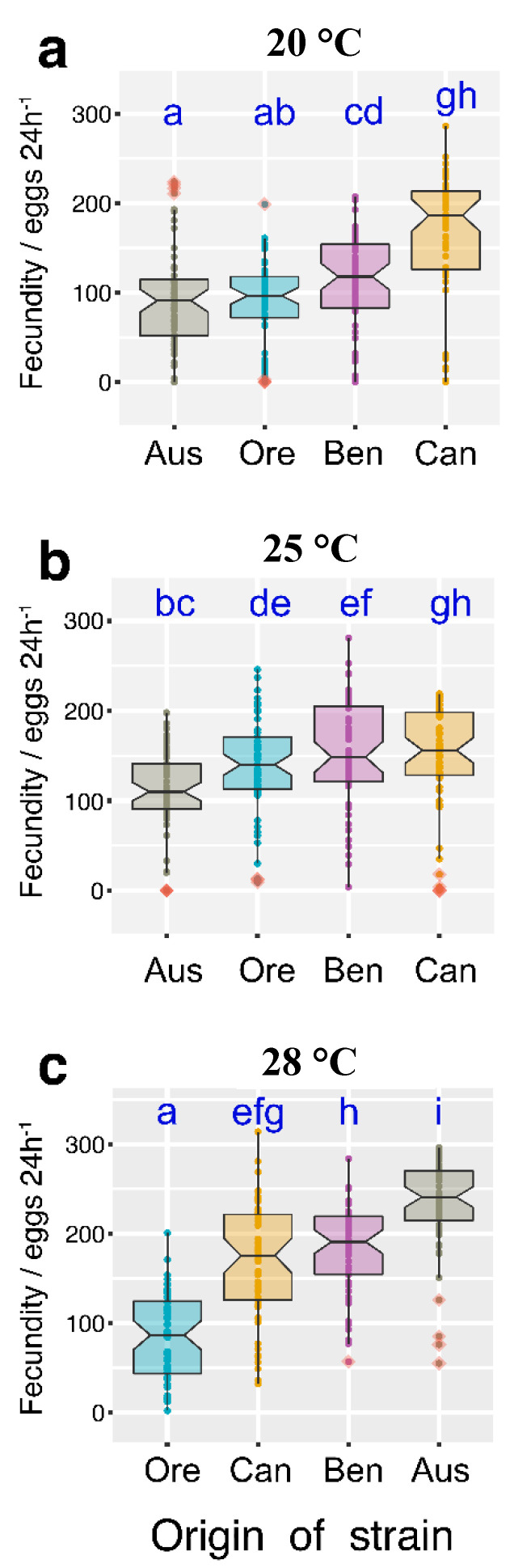
Effect of origin of strain and temperature on fecundity. Number of eggs laid by a female on a standard diet at 20 °C (**a**), 25 °C (**b**), and 28 °C (**c**). The box plots (median, interquartile range, and 95% confidence interval) describe the variability of the strains in the order of increasing median values. Robust two-way ANOVA (pbad2way function, R package *WRS2*) was followed by a robust post-hoc test (pairwise Robust Matrix function, *WRS2* package in R) for pairwise comparison of the median of groups. Statistical (*p* < 0.05) differences among the groups of various origin (across all the temperature levels) are indicated by different letters. The letters are alphabetically ordered according to the ascending median values. The outliers in the plot are marked by the shaded squares. The measurements are based on 10 replicate vials with 30 flies in each vial. Aus—Australian population; Ben—Benin population; Can—Canadian population; Ore—OregonR laboratory strain.

Temperature largely determines lifespan regardless of the genotype. Therefore, we subsequently examined whether the temperature would also affect other parameters of the fruit fly’s life such as fertility. Our data show that temperature had a significant effect on fertility expressed as the number of eggs laid within 24 h (*p* < 0.05) (Figure 3). In general, females laid more eggs at higher temperature. At 20 °C, the Canadian females laid the highest number of eggs, while females with other genotypes were at a statistically similar level of fertility (Figure 3a) (*p* < 0.05). At 25 °C and 20 °C, we observed the highest fertility in the Canadian female’s genotype and the lowest in the Australian females (Figure 3a,b) (*p* < 0.05). At 28 °C, females from Australia had the highest fertility. Females from Benin and Canada had significantly lower fertilities, while we observed the lowest fertility in the OregonR females (Figure 3c) (*p* < 0.05).

### 3.3. Temperature Effect on Body Weight

**Figure 4:**
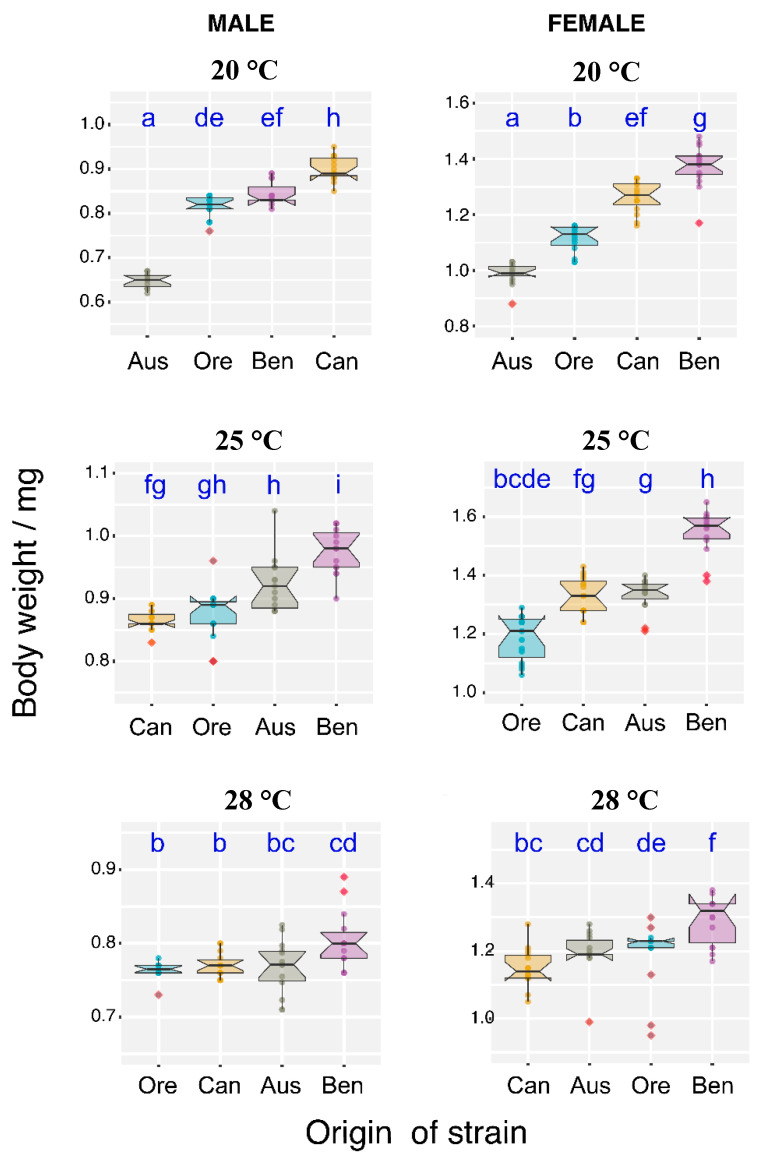
Effect of temperature on the body weight in male (A) and female (B) flies on a standard diet at 20 °C, 25 °C, and 28 °C. The box plots (median, interquartile range, and 95% confidence interval) describe variability of the strains in the order of increasing median values. Robust two-way ANOVA (pbad2way function, R package *WRS2*) was followed by a robust post-hoc test (pairwise Robust Matrix function, *WRS2* package in R) for pairwise comparison of the median of groups. Statistical (*p* < 0.05) differences among the groups of various origin (across all the temperature levels) are indicated by different letters. The letters are alphabetically ordered according to the ascending median values. The outliers in the plot are marked by the shaded squares. The measurements (10 replicate vials with 30 flies in each vial) and statistical analysis were performed separately for each sex.

At 25 °C, the body weights of males and females were the highest (with exception of the Canadian males and the OregonR and Canadian females) (*p* < 0.05). Generally, females were heavier than males. The mean body weight of males did not exceed 1 mg, while all females (except for the ones from Australia living at 20 °C) had a body weight well above 1 mg (Figure 4). In the case of females, the temperature had the highest impact on the Australia and Benin genotypes (Figure 4b,d,f). Females from Benin had the highest body weight at all analyzed temperatures (*p* < 0.05). The lowest temperature effect on body weight was observed for the OregonR strain females (*p* < 0.05). Males originating from Australia were the lightest at 20 °C, and the Canadian were the heaviest (*p* < 0.05). As seen in Figure 4b, males from Benin had the highest body weight, while the Canadian and OregonR males were the lightest (*p* < 0.05). At 28 °C, the body weight of males was the lightest compare with 20 °C and 25 °C (*p* < 0.05). 

### 3.4. Calorimetric Results

**Figure 5:**
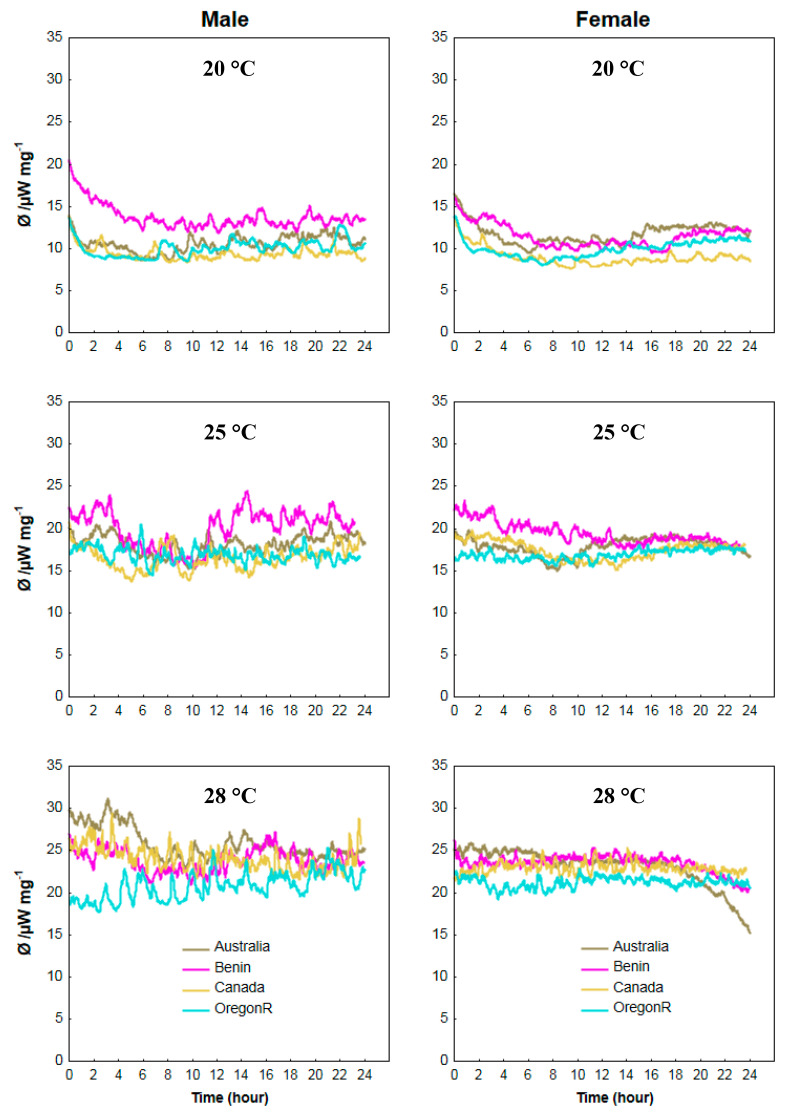
Specific thermal power: time curves representing the heat flow for male and female *Drosophila melanogaster* flies of different origin of strain and at different growth temperatures.

Changes in the metabolic heat produced by flies (ϕ), measured in microwatts and depending on the ambient temperature may be seen in Figure 1a–f. Analysis of the ϕ–t plots (µW mg^−1^) showed a significant effect of culture temperature on the metabolic heat flow of the flies. Both males and females were characterized by higher *ϕ* values at higher temperatures (Figure 5a–f). We therefore observed the lowest *ϕ* values in flies reared at 20 °C and the highest at 28 °C (Figure 5a,b,e,f). No significant differences in heat flow were found between males and females within the same temperature limits. However, the daily course of the ϕ–t plots was more varied for male genotypes than for female ones.

**Figure 6:**
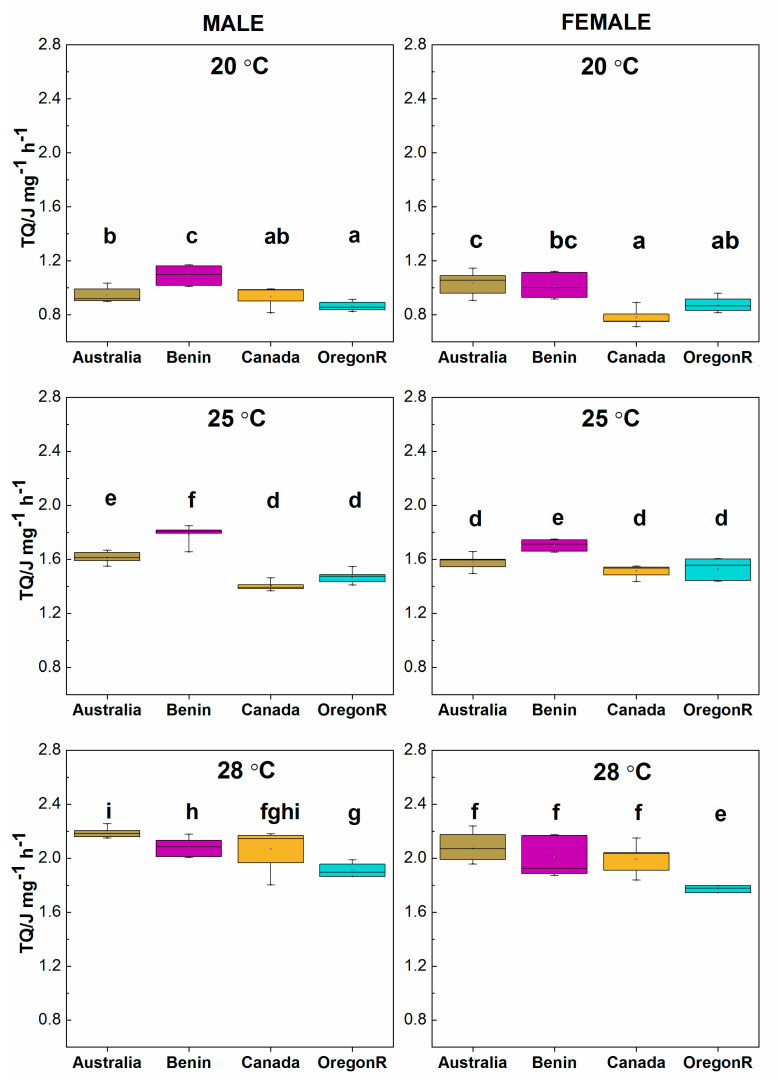
Total heat flow (TQ - metabolic activity in J∙mg^−1^∙h^−1^) for male and female flies of *Drosophila melanogaster* for three different growth temperatures and various origin of strain. Robust two-way ANOVA (pbad2way function, R package *WRS2*) was followed by a robust post-hoc test (pairwise Robust Matrix function, *WRS2* package in R) for pairwise comparison of the median of groups. Statistical (*p* < 0.05) differences among the groups of various origin (across all the temperature levels) are indicated by different letters. The letters are alphabetically ordered according to the ascending median values. The outliers in the plot are marked by the shaded squares. The measurements (10 replicate vials with 30 flies in each vial) and statistical analysis were performed separately for each sex.

For the Benin males, the *ϕ* values at 20 °C were significantly higher than for males from all other strains (Figure 5a) (*p* < 0.05). For the Benin females, we only found a higher thermal power emission at the initial stages of the measurement (between 2 and 6 h). Moreover, we found an increase of the *ϕ* value from hour 14 of the measurement for the Australia females’ strain (Figure 5b) (*p* < 0.05). At 25 °C, the highest *ϕ* values were observed for males and females from Benin. However, for males the ϕ–t curve fluctuated with the heat rate higher compared to other genotypes in the first 4 h and from hour 11 to the end of the 24 h cycle (Figure 5c) (*p* < 0.05). For the Benin females, the heat flow gently dropped until hour 11 of the cycle and was similar to the *ϕ* values of the other genotypes from this time point onward (Figure 5d) (*p* < 0.05). At 28 °C, we found the lowest values of heat flow for males and females of the OregonR strain (Figure 6e,f). The low survivorship of the OregonR genotype at 28 °C was confirmed by the data presented in Figure 2e,f. 

Mobility of the flies can be determined by the analysis of the ϕ–t curves [44]. Jagged time course of the curves for the male flies (Figure 5a,c,e) is explained by the flying activity of the flies in the ampoule during the measurement. On the other hand, the smoother course of the curves with less amplitude of changes for females indicates movement of the flies around the whole ampoule with no flying (Figure 5b,d,f). However, some increase in the activity and a tendency to fly during the first 12 h of the cycle can be observed for the female Benin genotype at 25 °C (Figure 5d).

The total heat flow (TQ) produced by males and females in all genotypes increased with increasing temperature from approximately 1.0 (J mg^−1^ h^−1^) at 20 °C to approximately 2.1 (J mg^−1^ h^−1^) at 28 °C (Figure 6a–f). 

**Figure 7:**
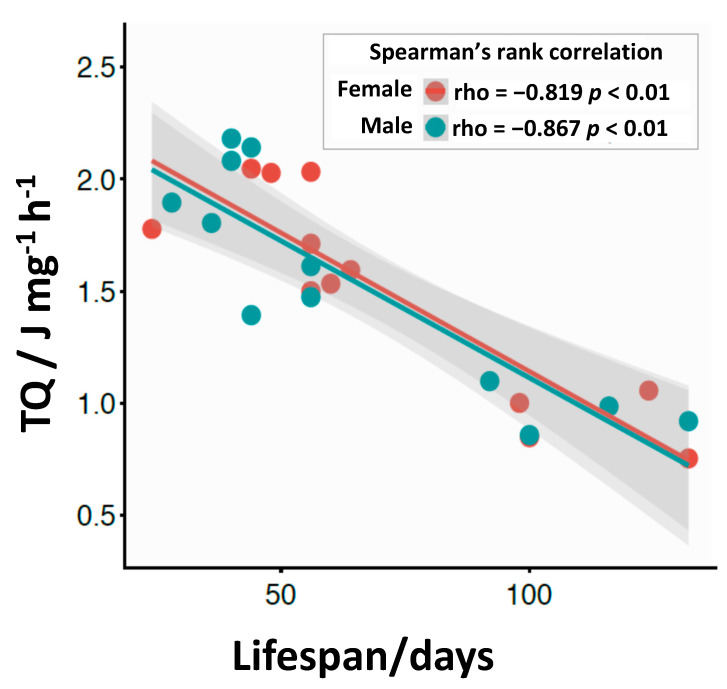
Scatter plots presenting the relationship between lifespan and total heat flow values (TQ - metabolic activity) for females and males of the *Drosophila melanogaster* fruit fly. The straight lines depict a linear trend in the data. The 95% confidence intervals are given as a shaded area. Spearman’s rank rho correlation coefficients for medians and *p*-value are also given.

The largest TQ at 20 °C and 25 °C was recorded for the Benin males and females (Figure 6a–d). At 28 °C, the OregonR males showed a significantly lower TQ (1.9 J mg^−1^ h^−1^), while the Australian strain showed the highest TQ (Figure 6e) (*p* < 0.05). As seen in Figure 6e, the OregonR females showed a significantly lower TQ (1.8 J mg^−1^ h^−1^), while for the other genotypes the TQ did not significantly differ (*p* < 0.05). Generally, a higher rearing temperature increased the metabolic activity of *D. melanogaster* (measured as the total heat flow—TQ) and, consequently, reduced survivorship of the flies. Survivorship for male and female genotypes can be prolonged by lowering their metabolic activity (elongation of straight line at 1.00 on the survivorship curves). A strong negative correlation between heat rate and mean lifespan is evident, although the tested fruit flies strongly differed in their geographic origin. Spearman’s rank rho correlation coefficient values were −0.819 for female and −0.867 for male *D. melanogaster* (*p* < 0.01) (Figure 7). 

## 4. Discussion

Aging is a universal process that occurs in all living organisms from the simplest bacteria to plants and animals [4]. It is interesting that despite many studies and much effort put into research on that complex process, there is no unambiguous answer to the following question: What is the main factor that lengthens or shortens the lifespan? So far, several important factors have been described as those that may significantly affect lifespan, yet not all mechanisms are thoroughly understood. Nevertheless, it is clear that the mechanism of aging may be modulated by environmental and genetic factors [45,46].

In this work, we have shown that significant reduction in the rate of metabolism is one of the universal mechanisms that can lead to longevity. One of the more vividly discussed factors modulating the rate of metabolism is temperature. According to van’t Hoff’s rule, an increase in temperature increases the rate of chemical reaction and the rate of chemical reaction increases exponentially with temperature. It seems, therefore, that temperature is one of the most important determinants of lifespan in poikilothermic organisms. Earlier works on the budding yeast as a model organism led to similar conclusions: a) temperature determines lifespan; and b) the rate of metabolism is the key to achieving longevity. First of all, temperature significantly modulates longevity in these unicellular fungi [30]. Moreover, genetic manipulations, such as protein biosynthesis disorders which lead to a reduction in the rate of metabolism, significantly extend the lifespan of yeast [21]. Therefore, in this study, we investigated whether the metabolic rate is the most important factor in determining the aging rate of the fruit fly originating from different continents.

For the purpose of the analysis, we used four fly strains originated from Australia, Africa (Benin), North America (Canada), and the OregonR laboratory strain [33,36,47,48]. We assumed that in nature, these strains live in different optimum temperatures [49]. Therefore, it was interesting to test their lifespans at different temperatures. Our lifespan analysis fully confirmed the earlier extensive data on the effect of temperature [50,51]. In general, the fruit fly is known to exhibit many genetics differences among geographic location. Additionally, there are some problems concerned with laboratory storage of wild populations. As was shown previously, a laboratory line subjected to a constant thermal regime for twelve years showed some thermal selection effects, e.g., higher size, short lifespan [32]. Interestingly, the threshold of cultured wild populations in a laboratory is unknown, which allowed for the use of wild populations without a thermal selection effect. In this study, we used fruit flies cultured in a laboratory over many years, but the results did not show, for example, that the Benin population reared fifty years ago had drastically impaired viability in higher temperatures or fecundity in all analyzed temperature. Furthermore, the Australian population reared ten years ago did not exhibit statistical significance in longevity with a temperature of 28 °C. However, those long-term populations cultured in a laboratory, in the case Benin population, influenced an increased body size which is in line with previous data [32]. 

More than a century ago, Loeb and Northrop [5] were the first to show that the fruit fly’s lifespan correlates negatively with temperature. Subsequent data showed that *D. melanogaster* lives longer at a low rather than high temperature [17,52]. These results were confirmed by Leiser et al. [50] who concluded that temperature had a significant effect on lifespan. Interestingly, a recent study has shown that inducing mild heat stress may also decrease the fruit fly’s aging rate by stimulating pathways associated with genome stability during hormesis [53]. Hormesis in aging positively supports life due to the cellular responses to single or multiple rounds of mild stress [54,55,56,57]. Temperature determines lifespan also in other invertebrates. Much attention was devoted to studies involving the nematode *Caenorhabditis elegans* [51]. As highlighted by Keil et al. [58], there is no invertebrate species in which longevity has been shown to increase with temperature. Indeed, many studies in insects noted a negative correlation between temperature and longevity [22,59,60,61]. In turn, use of some aphid species seems to contradict this general statement. The data show that a temperature increase leads to lifespan extension, which suggests that optimum temperature and genotype may be more important in the case of some species of insects [22,62]. Longevity effects during temperature manipulation were also demonstrated when using poikilothermic vertebrates, e.g., fish [63,64]. Munch and Salinas [65] highlighted the fact that there is a clear trend for longer lifespans at lower temperatures in wild species of ectotherms. Among homoeothermic vertebrates, the mouse was often examined [66]. In this study, we focused on *D. melanogaster* as an invertebrate model organism; further information on other invertebrates as well as poikilothermic and homoeothermic vertebrates has been extensively reviewed elsewhere [58]. Longevity resulting from decreased temperature is well known in both endothermic and exothermic species, but the mechanism underlying this change in aging is still poorly understood. In general, it is thought that low temperature decreases the metabolic rate thus slowing the rate of damage of macromolecules (nuclei acids, proteins, lipids) in cells caused by reactive oxygen species. Recently, studies have suggested that the mechanism of lifespan extension through low temperature is an active genetic process rather than a passive thermodynamic one and is dependent upon genotype. Additionally, a probable mechanism of lifespan extension by low temperature may work via non- or partially overlapping molecular pathways, e.g., by calorie restriction response [67]. Interestingly, in *Drosophila* short-term exposures to low temperatures lead to long-term increase in longevity and stress resistance, suggesting that it is not a short-term decrease in metabolism but rather a long-term physiological adaptation that controls lifespan [68].

In 1908, Max Rubner [19] announced the “rate of living” theory which assumes that energy consumption by different species of mammals during their lives based on the unit of volume is similar despite differences in life expectancy. Therefore, some mammals use energy at a quicker pace and live shorter, or they can consume it more economically which allows them to live for a longer period. In 1928, Pearl [69] expanded the theory and hypothesized that longevity is inversely proportional to the level of basic metabolism. The “rate of living” theory does not apply to mammals only as originally assumed which was confirmed by research using other animal groups, for example, insects *D. melanogaster* [17]. Our previous data with the use of the budding yeast also confirmed that the rate of metabolism is the major factor determining longevity [21].

Our results confirm the “rate of living” theory. To express the real rate of metabolism in specific temperatures, we used a unique method of isothermal calorimetry. This method allowed us to track the heat flow (metabolic heat production) over the time course of the experiment. Heat flow analyses are an important tool to estimate parameters that affect the actual rate of metabolism. As we showed, there is a very strong correlation between the total heat flow (TQ) and longevity. For males and females, the high rate of metabolism (high TQ) determines faster aging regardless of the genotype, and lower heat flow resulting from low level of metabolism leads to an increased lifespan. This suggests that the metabolic rate is a crucial factor in determining longevity. 

It should be mentioned that Pearl’s theory is not universally accepted. Speakman et al. [19] were the first to provide an important proof that within a population of mice there is a positive correlation between metabolic rate and longevity. These data are contrary to the theories of aging that inversely link energy expenditure to aging. Speakman et al. [19] proposed an explanation for why a positive correlation might exist, suggesting that the increased activation of uncoupling protein may decrease production of reactive oxygen species and increase the rate of metabolism. 

Attention was also focused on the influence of temperature on development. In general, a negative correlation was noted between the developmental temperature and body size [70]. Previous studies suggested that the decreased lifespan of flies reared at higher temperatures could be due to their small body size; conversely, a large body weight correlates with an increased lifespan [52,71,72]. Interestingly, Zwaan et al. [52] suggest that wing size is a better measure of adult body size than body weight, because larger variability was observed in the female body weight due to the egg production. In turn, our data showed that there was no correlation between longevity and body weight. In our analysis, the fruit fly achieved the largest body weight in the optimum temperature of 25 °C yet rearing them at 20 °C significantly increased the lifespan. Previous studies also noted a lack of positive correlation between lifespan and body size in various temperature groups [52] which supports our results. It should be noted that Zwaan et al. [52] demonstrated that fruit fly growth at 25 °C resulted in a longer lifespan than at 20 °C. It seems that the general metabolic activity rather than body weight generally determines longevity. Our results therefore contradict the earlier report that suggests that larger fruit flies have a lower energy expenditure per weight unit than the smaller ones [73]. Here, we demonstrated that larger fruit flies (growth at 25 °C) have higher TQ than the smaller ones (growth at 20 °C). Also, previous studies show that the respiration rate [74] and activity of antioxidant enzymes, such as superoxide dismutase and catalase [75], cannot explain the significant reduction of lifespan during growth at higher temperatures. 

To conclude, the investigations that we conducted using *D. melanogaster* of different origins suggest that genomic effects (assumed by the different sites of origin of the populations) may explain most of the observed effect variations—but not all. Although we observed some relationships between life expectancy and temperature, a clear geographic pattern was not observed. As shown by Trotta et al. [32], adult longevity was a trait for which there was no significant difference between tropical and temperate flies living in the experimental thermal range of 11 to 32 °C. Importantly. longevity in nature depends on season and place [76,77], and what is measured in the lab might be unrelated to fitness in the wild. Fly strain storage may also be problematic in research, but as was shown, adaptive responses were observed for the developmental rate, fertility, and body size but not for longevity [32]. It is possible that our results were affected by the long period of laboratory culture of our experimental populations, as it was previously emphasized [78]. Differences in lifespan were most pronounced between the sexes, irrespective of temperature or origin. These differences sharply decreased when flies were grown at higher temperatures. It is probable that both genome expression and temperature have an impact on lifespan; however, at high temperatures the genotype effect is not visible. It seems that a broad analysis of gene expression is necessary to understand the aging mechanisms that lead to longevity. It also seems important for the future of the research to show whether the loss of some wild traits in flies is the result of plasticity or accumulation of mutations resulting from the increasing homozygosity of the strains, as is the case with many laboratory lines.

## Supplementary Materials

The following are available online at https://www.mdpi.com/2075-4450/11/8/470/s1, Table S1: Lifespan of wild type *Drosophila melanogaster* flies of various origin cultured at different temperatures. Values mean respectively: median, mean ± standard deviation, minimum and maximum values (M—Male; F—Female).
