# Supplementary material for: Effects of Temperature on Lifespan of Drosophila melanogaster from Different Genetic Backgrounds: Links between Metabolic Rate and Longevity
Source: Insects. 2020 Jul 25;11(8):470. doi: 10.3390/insects11080470 (PMC7469197; doi:10.3390/insects11080470)
Supplement: Supplementary file 1 [file insects-11-00470-s001.pdf]

Table S1 Lifespan of wild type *Drosophila melanogaster* flies of various origin cultured at different temperatures. Values mean respectively: median, mean ± standard deviation, minimum and maximum values (M – Male; F – Female)

Temperature [°C]	Lifespan/days							
	**Med_M**	**Med_F**	**$Mean_M$**	**$Mean_F$**	**Min_M**	**Min_F**	**Max_M**	**Max_F**
				**Australia**				
**20**	132	124	123 ± 24	120 ± 23	4	8	148	152
**25**	56	68	52 ± 12	63 ± 16	1	4	80	88
**28**	40	44	37 ± 9	43 ± 15	8	4	48	64
				**Benin**				
**20**	92	98	94 ± 26	94 ± 37	16	4	152	152
**25**	44	60	38 ± 11	57 ± 11	12	16	60	72
**28**	40	48	36 ± 9	47 ± 9	4	24	48	60
				**Canada**				
**20**	116	132	106 ± 34	129 ± 28	4	4	148	164
**25**	44	64	40 ± 16	52 ± 26	1	1	72	88
**28**	44	56	41 ± 12	55 ± 10	4	16	60	68
				**OregonR**				
**20**	100	100	90 ± 31	106 ± 16	4	76	124	136
**25**	56	56	51 ± 15	52 ± 17	4	4	76	84
**28**	28	24	29 ± 6	26 ± 5	4	16	40	40
